# Oncolytic adenoviruses synergistically enhance anti-PD-L1 and anti-CTLA-4 immunotherapy by modulating the tumour microenvironment in a 4T1 orthotopic mouse model

**DOI:** 10.1038/s41417-021-00389-3

**Published:** 2021-09-24

**Authors:** Huan Zhang, Weimin Xie, Yuning Zhang, Xiwen Dong, Chao Liu, Jing Yi, Shun Zhang, Chunkai Wen, Li Zheng, Hua Wang

**Affiliations:** 1 Department of Breast, Bone and Soft Tissue Oncology, The Affiliated Tumour Hospital of Guangxi Medical University, Nanning, P.R. China; 2 Department of Experimental Haematology, Beijing Institute of Radiation Medicine, Beijing, P.R. China; 3 Department of Experimental Medical Science & Key Laboratory of Diagnosis and Treatment of Digestive System Tumours of Zhejiang Province, HwaMei Hospital, University of Chinese Academy of Sciences, Ningbo, Zhejiang PR China; 4 State Key Laboratory of Proteomics, Beijing Proteome Research Center, National Center for Protein Sciences, Beijing Institute of Lifeomics, Beijing, P.R. China

**Keywords:** Cancer immunotherapy, Breast cancer

## Abstract

Effective therapeutic strategies for triple-negative breast cancer (TNBC) are still lacking. Clinical data suggest that a large number of TNBC patients cannot benefit from single immune checkpoint inhibitor (ICI) treatment due to the immunosuppressive tumour microenvironment (TME). Therefore, combination immunotherapy is an alternative approach to overcome this limitation. In this article, we combined two kinds of oncolytic adenoviruses with ICIs to treat TNBC in an orthotopic mouse model. Histopathological analysis and immunohistochemistry as well as multiplex immunofluorescence were used to analyse the TME. The immunophenotype of the peripheral blood and spleen was detected by using flow cytometry. Oncolytic adenovirus-mediated immune activity in a coculture system of lytic supernatant and splenocytes supported the study of the mechanism of combination therapy in vitro. Our results showed that the combination of oncolytic adenoviruses with anti-programmed cell death-ligand 1 (anti-PD-L1) and anti-cytotoxic T lymphocyte-associated antigen-4 (anti-CTLA-4) (aPC) can significantly inhibit tumour growth and prolong survival in a TNBC model. The combination therapy synergistically enhanced the antitumour effect by recruiting CD8^+^ T and T memory cells, reducing the number of regulatory T cells and tumour-associated macrophages, and promoting the polarization of macrophages from the M2 to the M1 phenotype to regulate the TME. The rAd.GM regimen performed better than the rAd.Null treatment. Furthermore, aPC efficiently blocked oncolytic virus-induced upregulation of PD-L1 and CTLA-4. These findings indicate that oncolytic adenoviruses can reprogramme the immunosuppressive TME, while ICIs can prevent immune escape after oncolytic virus therapy by reducing the expression of immune checkpoint molecules. Our results provide a mutually reinforcing strategy for clinical combination immunotherapy.

## Background

Triple-negative breast cancer (TNBC) is the leading cause of metastasis and death among females, and there are no effective therapeutic strategies because of its genomic instability and high mutation rate. Immunotherapy, rapidly developed as a tremendously promising approach for cancer treatment, may open a new chapter in the treatment of TNBC [1, 2].

Immune checkpoint inhibitors (ICIs) targeting programmed cell death-1 (PD-1)/programmed cell death-ligand 1 (PD-L1) or cytotoxic T lymphocyte-associated antigen-4 (CTLA-4) have become one of the most impressive immunotherapies because of their potent and durable therapeutic efficacy [3, 4]. However, their clinical efficacy is greatly hindered by the depletion of killer T cells and the recruitment of immunosuppressive T cells in the tumour microenvironment (TME) [5, 6]. The lack of tumour-infiltrating lymphocytes and chemokines for T cell recruitment significantly reduces the antitumour effects of ICIs, especially in poorly immunogenic tumours, such as renal carcinoma, TNBC, cervical cancer and glioma [7, 8]. Previous clinical trials have suggested that single ICI therapy produces limited antitumour responses in TNBC patients [9, 10]. Therefore, additional therapies are needed to increase the sensitivity of tumour cells to ICIs, such as activating and recruiting immune cells [6, 11].

Oncolytic viruses have emerged as novel weapons in the ‘war’ of immunotherapy because selective replication and direct oncolysis in tumour cells are coupled with successful elicitation of antitumour immunity [12, 13]. Nevertheless, the therapeutic efficacy of oncolytic viruses was confined due to an ‘immune brake’ in the TME [14]. It has been widely demonstrated that oncolytic viruses can recruit immune cells to the TME in a TNBC model. However, these viruses can also upregulate the expression of PD-L1 on breast cancer cells and in turn result in immune escape [15, 16]. Therefore, it is of great significance to find a combination therapy that can relieve the ‘immune brake’ to maximize the immunotherapeutic efficacy of oncolytic viruses [17].

Thus, the combination of oncolytic viruses and ICIs may be a reasonable and promising strategy to synergistically overcome immunosuppression in the TME.

Granulocyte-macrophage colony stimulating factor (GM-CSF) could promote the immune activation effect of the oncolytic virus [18, 19]. Our laboratory has previously developed oncolytic viruses carrying the GM-CSF gene, named rAd.GM. In this study, we established a TNBC orthotopic model in immune-competent BALB/c mice. We showed that the antitumour effects of anti-PD-L1 and anti-CTLA-4 (aPC) were enhanced by combining with rAd.GM through modulation of the TME. In addition, aPC hindered immune escape after oncolytic virus therapy. Our investigations provide a rational combination strategy for the treatment of TNBC.

## Materials and methods

### Cell lines

The mammary tumour cell lines 4T1, EMT-6 and MDA-MB-231 were purchased from ATCC and Procell. 4T1 and MDA-MB-231 cells were cultured in Dulbecco’s modified Eagle medium (Gibco, NY, USA) supplemented with 10% foetal bovine serum (FBS) (Gemini, NY, USA). EMT-6 cells were cultured in Roswell Park Memorial Institute (RPMI) 1640 (Gibco, NY, USA) supplemented with 10% FBS.

### Oncolytic adenoviruses

The oncolytic adenoviruses were constructed by using a simplified system for generating oncolytic adenovirus vectors carrying one or two transgenes as reported previously. The replication of adenoviruses is controlled by TERTp, located upstream of E1A. For rAd.GM, the expression of human GM-CSF and E1B55K was linked by a ribosomal internal entry site and initiated by an E1B promoter. rAd.Null is a control oncolytic adenovirus devoid of any foreign transgene. The viruses were amplified in HEK293 cells and purified by density gradient centrifugation with caesium chloride.

### rAd.GM-mediated GM-CSF secretion in the culture medium

4T1 cells were plated into a 6-well plate (3 × 10^5^ cells/well). Eight hours later, the cells were infected with 2000 viral particles (VPs)/cell of rAd.GM. Four hours after infection, the cells were washed with phosphate-buffered saline (PBS) and transferred to fresh medium, and then the incubation was continued for 44 h. The GM-CSF secreted in the supernatant was quantified by an enzyme-linked immunosorbent assay kit according to the manufacturer’s instructions (NEOBIOSCIENCE, Wuhan, China).

### Animals

Female BALB/c mice between 6 and 8 weeks of age were purchased from Beijing Vital River Laboratory Animal Technology Co, Ltd. (Beijing, China). They were housed in a specific pathogen-free animal facility within a stable environment (temperature 20–24 °C, humidity 45–65% and 12-h light–dark cycles). All procedures for the animal experiments were approved by the Institutional Animal Care and Use Committee (IACUC) at the Beijing Institute of Radiation Medicine (IACUC-DWZX-2020-669).

### Tumour models and treatment regimens

To establish TNBC models, 6.0 × 10^5^ 4T1 cells were injected (day 0) into the number 3 and 4 mammary fatty pads of BALB/c mice. When tumours were visible (on day 7 after cell injection), tumour volumes were measured by callipers and calculated using the following formula: tumour volume = width^2^ × length/2.

Then tumour-bearing mice were randomly divided into five or six groups: control group, rAd.Null, rAd.GM, aPC, rAd.GM + aPC, and rAd.Null+aPC group (we did establish the rAd.Null + aPC group until we observed the overall therapeutic effect). On day 7, rAd.Null or rAd.GM (2.5 × 10^10^ VPs of each virus in 100 µL PBS) or PBS (100 µL) was administered directly into the tumours. A repeat viral dose was given on day 10. On days 8, 11 and 14, 10 mg/kg atezolizumab (anti-PD-L1) (Lot 20190801A) and 4 mg/kg ipilimumab (anti-CTLA-4) (Lot 20200214) (Kohnoor, Beijing, China) were administered intraperitoneally. On day 24, the mice were euthanized, tumours were removed and weighed and other samples were collected. The animals that survived were maintained until natural death.

### Immunophenotype analysis of the peripheral blood and spleen

On days 8, 16, 21 and 24, peripheral blood samples were collected and lysed by RBC lysis buffer. On day 24, spleens were collected, and splenocytes were isolated and lysed with RBC lysis buffer. Single-cell suspensions were stained with panel 1: PE-anti-mCD3 antibody (Lot 05122-60-100), FITC-anti-mCD4 (Lot 06122-50-100), and APC-anti-mCD8 (Lot 10122-80-100); panel 2: FITC-anti-mCD4 (Lot 06122-50-100), APC-anti-mCD25 (Lot 07312-80-100), and PE-anti-mFoxP3 (Lot 83422-60-100); panel 3: FITC-anti-mCD4 (Lot 06122-50-100), APC-anti-mCD44 (Lot 06511-80-100), and PE-cy7-anti-mCD62L (Lot 04712-77-100); and panel 4: FITC-anti-mCD8 (Lot 10122-80-100), PE-anti-mCD197 (Lot 20012-60-100), PE-cy7-anti-CD62L (Lot 04712-77-100), and APC- anti-mCD44 (Lot 06511-80-100). The immune phenotypes of T lymphocytes, including CD4^+^ T, CD8^+^ T, CD4^+^ regulatory T (Treg), memory T and T_IE_ cells, were analysed by flow cytometry. Various antibodies and other reagents for flow cytometry were purchased from BioGems (CA, USA).

### Histopathological analysis and immunohistochemistry (IHC)

On day 24, tumour and lung tissues were harvested, processed and stained with haematoxylin and eosin (H&E). Anti-mouse caspase-3 (CST, BSN, USA, #9662S) was used to detect caspase-3 expression.

### Multiplex immunofluorescence staining and imaging

Multiplex IHC staining was performed on 4-mm-thick, formalin-fixed, paraffin-embedded slides using an Opal multiplex IHC system (NEL811001KT, PerkinElmer) according to the manufacturer’s instructions. Briefly, after slide preparation and heat-induced epitope retrieval, slides were blocked with PerkinElmer Antibody Diluent Block buffer. The following primary antibodies were used: for panel 1, anti-CD4 (#25229), anti-CD25/IL-2Rα (#12653), anti-FOXp3 (#12653), anti-CD44 (#37259) (CST, BSN, USA), anti-CD8 (ab217344), and anti-CD62L (ab264045) (Abcam, Cambridge, UK); for panel 2, anti-CD68 (#97778), anti-CD16 (#73741), anti-CD274/PD-L1 (#64988) (CST, BSN, USA), anti-CD163 (ab182422), and anti-CD152L/CTLA-4 (ab237712) (Abcam, Cambridge, UK). After blocking, the sections were incubated with primary antibodies for 1 h at room temperature. After rinsing, the slides were incubated with polymer horseradish peroxidase-conjugated antibody specific to mouse or rabbit based on the primary antibody for 10 min at room temperature. After washing, Opal Fluorophore Working Solution was added to each slide. The slides were incubated at room temperature for 10 min to generate Opal signals. The stained slides were placed into an Opal slide-processing jar with antigen retrieval buffer and heated in a microwave. The primary and secondary antibodies were stripped from the slides and stained to detect the next target protein. After staining for all target proteins, cell nuclei were stained with 4′,6-diamidino-2-phenylindole (PerkinElmer, Waltham, USA), and images were taken using a Vectra Polaris automated quantitative pathology system. The images were analysed by the inForm 2.3.0 software (PerkinElmer, Waltham, USA).

### Oncolytic adenovirus-mediated immune activity in a coculture system of lytic supernatant and splenocytes

We used the abovementioned method to infect 4T1, EMT-6 and MDA-MB-231 cells with oncolytic viruses, and cells and supernatant were collected separately.

4T1 and EMT-6 cells were stained with PE-anti-mCD274 (PD-L1) (Lot 50-1243) (Tonbo Biosciences, CA, USA). In addition, spleens of normal female BALB/c mice were collected, splenocytes were isolated and the cell concentration was adjusted to 2 × 10^6^ cells/mL with RPMI 1640 medium containing 10% serum and added to 6-well plates at 1 mL per well. One millilitre of lysate supernatant/well was added to the coculture system. On day 3, single-cell suspensions were stained with panel 1: PE-anti-mCD3 (Lot 05122-60-100), FITC-anti-mCD4 (Lot 06122-50-100), and APC-anti-mCD8 (Lot 10122-80-100) and panel 2: PerCP Cy5.5-anti-mCD3 (Lot 05122-60-100) and PE- anti-mCD279 (PD-1) (Lot 31812-60-100) (BioGems, CA, USA).

MDA-MB-231 cells were stained with PE-anti-hCD274 (PD-L1) (Lot 329705) (Biolegend, CA, USA). Human lymphocytes were isolated from peripheral blood by lymphocyte separation medium (TBD, Tianjin, China) to construct a coculture system following the same steps as mentioned above. On day 3, single-cell suspensions were stained with PE-conjugated anti-hCD8 (Lot 344705) (Biolegend, CA, USA).

### mRNA expression of various genes

Total RNA was isolated from the other fraction of splenocytes and lymphocytes after coculture or tumour samples at terminal time points, and cDNA was synthesized using the StarScript II First-strand cDNA Synthesis Mix with gDNA Remover (GenStar, Beijing, China) according to the manufacturer’s instructions. The mRNA expression of T helper type 1 (Th1) cytokines, Th2 cytokines, chemokines and cytotoxicity-related genes was quantified by real-time reverse transcription PCR (RT-PCR) using RealStar Green Fast Mixture with ROX II (GenStar, Beijing, China) on an ABI 7500 fast system (Applied Biosystems, Thermo Fisher Scientific, CA, USA). The expression of the target genes was normalized to mouse β-actin expression. The detailed gene ID and primers’ sequences are shown in Table 1.

**Table 1:** The gene ID and primers’ sequences used in RT-PCR experiments.

Gene	Gene ID	Direction	Sequence (5’−3’)
Actin—Mouse	11461	Forward	AGGCCAACCGTGAAAAGATG
		Reverse	TGGCGTGAGGGAGAGCATAG
IL-2—Mouse	16183	Forward	TGAGTGCCAATTCGATGA
		Reverse	AGGGCTTGTTGAGATGATGC
IL-10—Mouse	16153	Forward	AGTGGTATAGACAGGTCTGTTGG
		Reverse	GCAGCTCTAGGAGCATGTGG
IFN-γ—Mouse	15978	Forward	ACTGGCAAAAGGATGGTG
		Reverse	GTTGCTGATGGCCTGATT
CCL5—Mouse	20304	Forward	ATATGGCTCGGACACCAC
		Reverse	GTGACAAACACGACTGCAA
Granzyme B—Mouse	14939	Forward	TGAAGTCAAGCCCCACTC
		Reverse	TCAGCACAAAGTCCTCTCG
Perforin—Mouse	18646	Forward	CTGGGATGCCGACTACG
		Reverse	CACCCTGCCGTGGTTTA
CXCL10—Mouse	15945	Forward	TTTCTGCCTCATCCTGCT
		Reverse	CCCTATGGCCCTCATTCT
TGF-β—Mouse	21803	Forward	CTCCCGTGGCTTCTAGTGC
		Reverse	GCCTTAGTTTGGACAGGATCTG
Actin—Homo	6774	Forward	GGACTGAGCATCGAGCA
		Reverse	GCCAGACCCAGAAGGAG
IL-2—Homo	3558	Forward	AGAACTCAAACCTCTGGAGGAAG
		Reverse	GCTGTCTCATCAGCATATTCACAC
IL-10—Homo	3586	Forward	ACCAAGACCCAGACATCAA
		Reverse	CATTCTTCACCTGCTCCAC
Granzyme B—Homo	3002	Forward	CCAGGGCATTGTCTCCTA
		Reverse	GGGGCTTAGTTTGCTTCC

### Statistical analysis

Statistical analyses were performed using GraphPad Prism (GraphPad Software). Values are represented as the mean ± SEM. Statistical differences were determined using one-way analysis of variance (ANOVA) followed by Bonferroni post hoc tests. The level of statistical significance was set at two-sided *p* < 0.05.

## Results

### Combination therapy effectively inhibited tumour growth and prolonged survival time in an immune-competent 4T1 orthotopic model

**Fig. 1:**
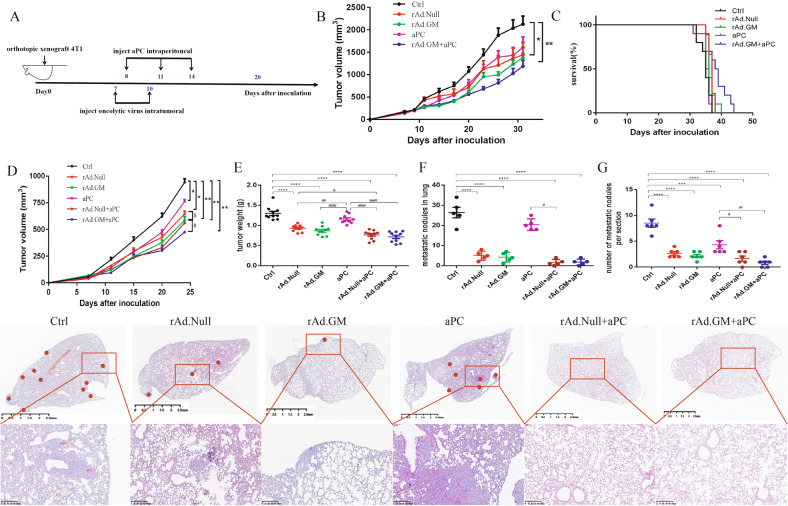
The combination therapy induces effective tumour growth inhibition and long-term survival benefit in 4T1 breast cancer. **A** Mice were treated as shown in the scheme. **B** Tumour volume of different treatment groups. **C** Kaplan–Meier plot for overall survival. **D** Comparisons of tumour growth curves over time. At the end of the experiment, intact tumours and lungs were removed. **E** Tumour weight (*n* = 10 for each group). **F** Tumour lesions in the lungs were counted (*n* = 5 in each group). **G** Lung slices were subjected to H&E staining to confirm metastasis. The statistical chart and representative images are shown (*n* = 6 for each group). The data are presented as mean ± SEM. *p < 0.05, **p < 0.01, ***p < 0.001, ****p < 0.0001 versus control; ^#^*p* < 0.05, ^##^*p* < 0.01, ^####^*p* < 0.0001 versus aPC; ^&^*p* < 0.05 versus corresponding oncolytic viruses. ^$^*p* < 0.05 rAd.GM versus rAd.Null. One-way ANOVA followed by Bonferroni post hoc tests was used.

To observe the potential therapeutic effects of aPC, oncolytic viruses and combinational therapy, we established an orthotopic 4T1 breast cancer model. aPC and oncolytic viruses were injected when the tumour volume reached approximately 100 mm^3^ (Fig. 1A). The results showed that aPC treatment inhibited tumour growth and slightly prolonged survival compared with the control, while the effects of the oncolytic viruses were modest. The combined therapy of aPC and rAd.GM produced much more impressive responses than others. The tumour volume was reduced by approximately 50%, and the survival time was prolonged by 1 week compared with the effects of the control (Fig. 1B, C).

To investigate the effects of combination therapy on tumour metastasis, we treated the orthotopic 4T1 breast cancer model with aPC, oncolytic viruses and combination therapy. We detected the tumour volume at different time points after 4T1 inoculation and weighed the tumours at the endpoint of the experiment. Consistent with the above experiments, the tumour volume (Fig. 1D) and tumour weight (Fig. 1E) were significantly reduced in the oncolytic virus and oncolytic virus combination groups, but the aPC group delayed tumour growth slightly. Importantly, rAd.GM combination therapy showed a greater enhanced effect than the rAd.Null combination therapy. At the end of the experiment, we removed the intact lungs and analysed tumour lung metastasis (Fig. 1F). In addition, we calculated metastatic lesions in lung sections by H&E staining (Fig. 1G). We found that both the oncolytic viruses and combination treatments effectively inhibited pulmonary metastasis, but only the combined treatments remarkably prevented metastasis, with these mice showing zero foci in approximately 20% of the lungs.

### Combination therapy promoted tumour cell apoptosis and necrosis

**Fig. 2:**
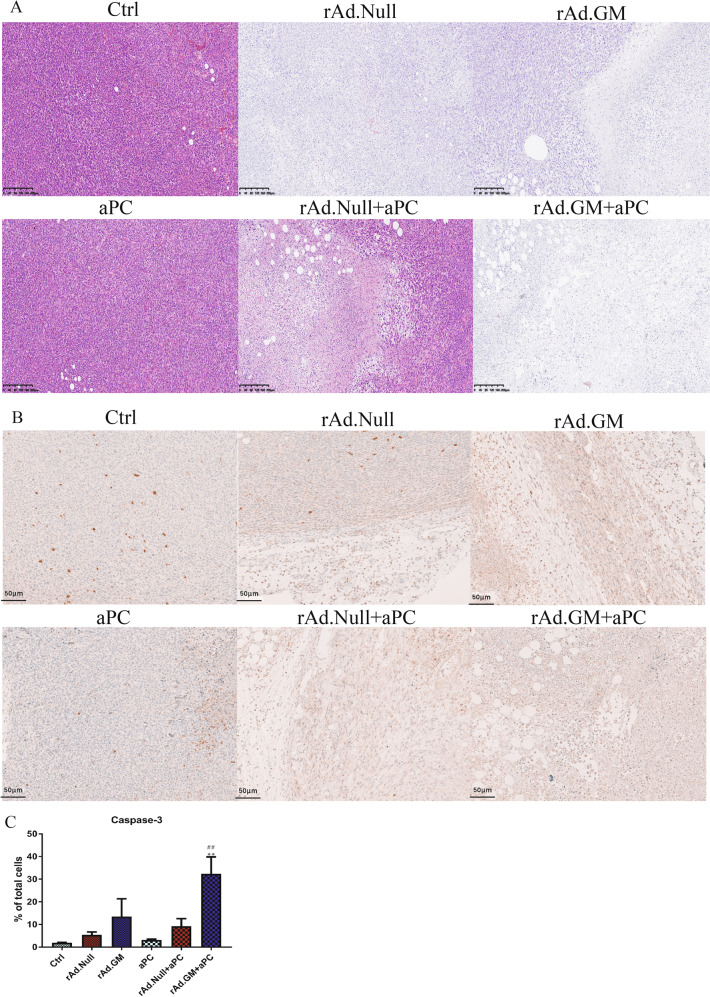
Combination therapy promoted apoptosis and necrosis in the TME. **A** The tumour tissue was subjected to H&E staining to confirm necrosis. **B** The tumour tissue was also subjected to immunohistochemistry to detect caspase-3 expression, and representative images are shown (scale bars = 50 μm). **C** The percentage of caspase-3^+^ cells. The data are presented as mean ± SEM. **p < 0.01 versus control; ^##^*p* < 0.01 versus aPC. One-way ANOVA followed by Bonferroni post hoc tests was used.

We next focused on apoptosis and necrosis in the local tumour. Histopathological analysis and IHC results indicated that all five treatment groups promoted tumour cell apoptosis and necrosis. The effect of oncolytic viruses, especially rAd.GM, was superior to aPC (Fig. 2A, B). Furthermore, the combination treatment group expressed more caspase-3 than the corresponding oncolytic virus groups, indicating that obvious apoptosis was induced. Importantly, the rAd.GM combination treatment group showed a higher apoptosis rate than the rAd.Null combination group (Fig. 2C).

### Combination therapy reprogrammes the TME by regulating lymphocyte infiltration and macrophage polarization

**Fig. 3:**
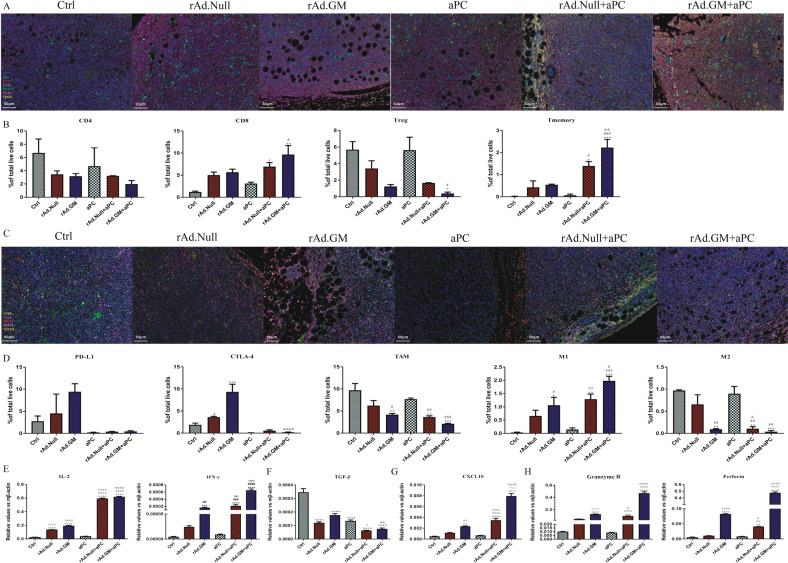
Combination therapy regulated lymphocyte infiltration as well as TAM polarization and immune-related genes in the TME. **A** Panel 1 of the multiplex immunohistochemistry detection of T cell infiltration in 4T1 tumour tissue (scale bars = 50 μm). **B** The percentages of CD4^+^ and CD8^+^ T cells, Tregs and memory T cells. **C** Panel 2 of the multiplex immunohistochemistry detection of immune checkpoint expression and macrophage polarization. **D** The percentages of CD274^+^ (PD-L1) and CD152L^+^ (CTLA-4) cells, TAMs and M1 and M2 macrophages. **E** On day 24, tumours were removed, and total RNA was isolated. After cDNA was synthesized, the expression of Th1 cytokines (IL-2 and INF-γ), Th2 cytokines (TGF-β), chemokines (CXCL10) and cytotoxicity-related genes (granzyme B and perforin) was analysed by real-time RT-PCR and normalized to β-actin (*n* = 4/group). The data are presented as mean ± SEM. *p < 0.05, **p < 0.01, ***p < 0.001, ****p < 0.0001 versus control; ^#^*p* < 0.05, ^##^*p* < 0.01, ^###^*p* < 0.001, ^####^*p* < 0.0001 versus aPC; ^&^*p* < 0.05, ^&&^*p* < 0.01, ^&&&^*p* < 0.001, ^&&&&&^*p* < 0.0001 versus corresponding oncolytic viruses. One-way ANOVA followed by Bonferroni post hoc tests was used.

To further explore the immune activation within the TME, multiplex immunofluorescence staining was used to observe the infiltrated lymphocytes. The results (Fig. 3A, B) showed that the combination therapy significantly upregulated CD8^+^ and reduced CD4^+^ T cells, increased the proportion of memory T cells and inhibited the infiltration of Tregs. Moreover, rAd.GM showed stronger effects on immune activation than rAd.Null in both the single-treatment groups and combination groups.

In addition, multiplex immunofluorescence staining results indicated that rAd.GM promoted macrophage polarization to the M1 phenotype, while aPC treatment had nearly no significant effect on the proportion of tumour-associated macrophages (TAMs) and macrophage polarization. Combination therapy decreased the number of TAMs in the TME and reprogrammed TAMs to polarize macrophages from the M2 to the M1 phenotype. These results indicate that the combination of rAd.GM and aPC not only inhibited the recruitment of TAMs but also promoted macrophage polarization from the M2 to M1 phenotype. In addition, we observed that the expression of CTLA-4 and PD-L1 was upregulated in the oncolytic virus-treated group but was eased when combined with aPC (Fig. 3C, D).

### Combination therapy regulated the Th1/Th2 balance and increased the expression of cytotoxicity-related genes in local tumour tissues

To further understand the mechanism of combination therapy, RT-PCR was used to detect the expression of inflammatory cytokines, chemokines and cytotoxicity-related genes in local tumour tissue (Fig. 3E–H). Oncolytic viruses and combination treatments, but not aPC treatment, obviously increased the expression of Th1 cytokines, which can evoke antitumour immune responses. The combination groups showed a much more remarkable increase in Th1 cytokines. Moreover, the rAd.GM and rAd.GM + aPC groups upregulated the expression of Th1 cytokines more significantly than the rAd.Null and rAd.Null+aPC groups, respectively (Fig. 3E). Th2 cytokines were downregulated in all five therapeutic groups, and the combined groups had a stronger effect than the oncolytic virus and aPC groups (Fig. 3F). The expression of chemokine CXCL10, a factor that represents the ability to recruit immune cells, was significantly upregulated only in the rAd.GM group among all the monotherapy groups, but it was remarkably higher in the combination treatment groups than in the rAd.GM group. As expected, the combination treatment with rAd.GM was stronger than the rAd.Null treatment (Fig. 3G). We also found that oncolytic viruses but not aPC promoted the expression of granzyme B and perforin, two major cytotoxic proteins released from natural killers and cytotoxic T lymphocytes. Combined therapy significantly enhanced the expression of these two genes, especially in the rAd.GM + aPC group, in which they were expressed fivefold higher than that of the rAd.Null+ aPC group (Fig. 3H).

### Oncolytic viruses enhanced the antitumour immune response of aPC by increasing CD8^+^ T cells and T memory cells and downregulating Treg cells in peripheral blood

**Fig. 4:**
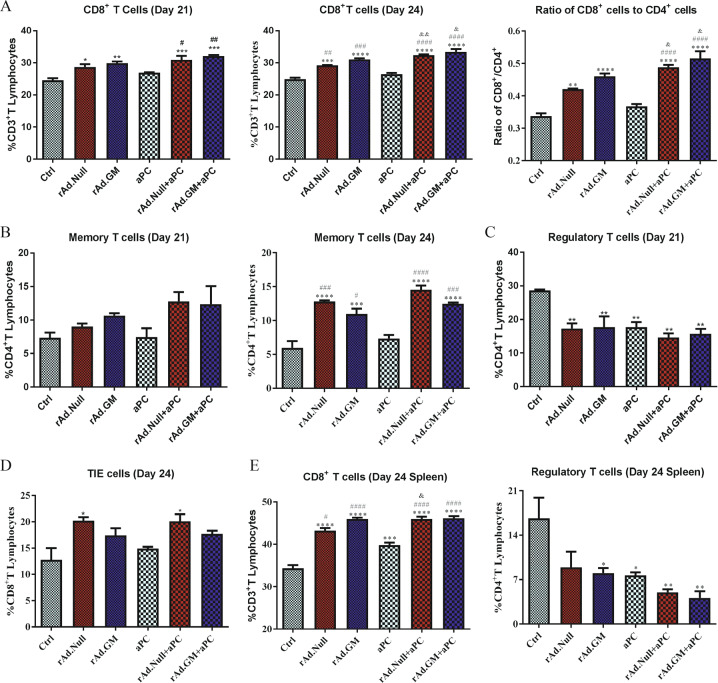
Oncolytic viruses improved the antitumour immune response of aPC by increasing the percentages of CD8^+^ T cells and memory T cells and reducing the percentage of Treg cells in peripheral blood and splenocytes. On days 8, 16, 21 and 24, peripheral blood cells were collected, and **A** the percentage of CD8^+^ T cells on days 21 and 24 as well as the ratio of CD8^+^ T cells to CD4^+^ T cells on day 24, **B** the percentage of memory T cells on days 21 and 24, **C** the percentage of Tregs on day 21 and **D** the percentage of T_IE_ cells on day 24 were analysed by flow cytometry. At the endpoint, spleens were collected, and the immune phenotypes of the splenocytes were analysed as previously described. **E** The percentages of CD8^+^ T cells and Tregs at day 24 are shown. The data are presented as mean ± SEM. *p < 0.05, **p < 0.01, ***p < 0.001, ****p < 0.0001 versus control; ^#^*p* < 0.05, ^##^*p* < 0.01, ^###^*p* < 0.001, ^####^*p* < 0.0001 versus aPC; ^&^*p* < 0.05, ^&&^*p* < 0.01 versus corresponding oncolytic viruses. One-way ANOVA followed by Bonferroni post hoc tests was performed.

CD8^+^ T cells are the major cell type that mediates the antitumour effect; therefore, we collected peripheral blood on days 8, 16, 21 and 24 and analysed the percentage of CD8^+^ T cells with fluorescence-activated cell sorter. On day 21, the oncolytic virus groups and combined treatment groups showed an increased percentage of CD8^+^ T cells, and this tendency was sustained until day 24. This effect was not seen in the aPC group. The ratio of CD8^+^ T cells to CD4^+^ T cells was analysed on day 24, and the results indicated that CD8^+^ T cells are the main components of specific antitumour immunity in vivo (Fig. 4A). The results also indicated that the percentage of CD4^+^CD44^+^CD62L^high^ T memory cells in the oncolytic virus groups and combined treatment groups was upregulated on days 21 and 24, and the percentage of CD4^+^ T memory cells in the combined treatment groups was higher than that of the corresponding oncolytic virus groups and aPC group (Fig. 4B). Combined treatment also downregulated the percentage of CD25^+^FoxP3^+^ Tregs among CD4^+^ T lymphocytes compared to the oncolytic virus groups and aPC group (Fig. 4C). T_IE_ cells, a kind of cytotoxic memory effector peripheral T cell or immune effector cell, play important roles in antitumour immunity. We also detected the expression of T_IE_ cells in peripheral blood (Fig. 4D) on day 24. We found that, compared with the control and aPC groups, the oncolytic virus and combined treatment groups showed an increased percentage of T_IE_ cells in peripheral blood.

### Oncolytic viruses enhanced the antitumour immune response of aPC in splenocytes

Immune activation in the spleen was also analysed on day 24. The results indicated that oncolytic viruses increased CD8^+^ T cells and downregulated Treg cells in splenocytes, while the combined treatment groups showed a greater effect than the oncolytic virus groups, which is similar to peripheral blood (Fig. 4E). These outcomes were consistent with the transformation of immune cell subsets in the TME.

### Immune activation effect of oncolytic virus therapies in vitro

**Fig. 5:**
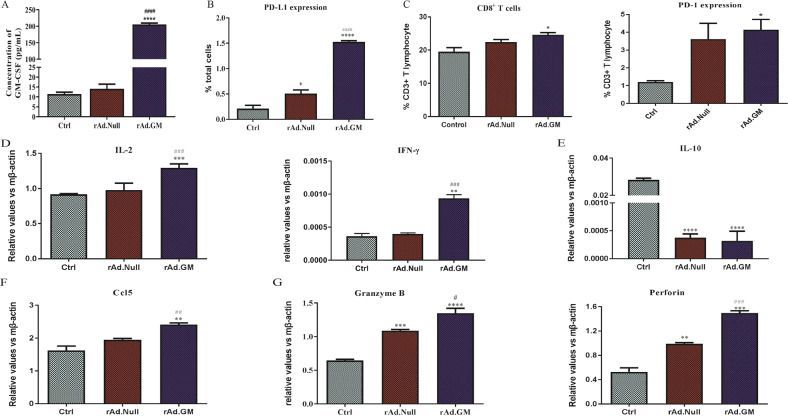
Immune activation effect of oncolytic virus therapies in vitro. 4T1 cells were infected with oncolytic viruses for 48 h, and the cells and supernatant were collected separately. **A** The secretion of GM-CSF in the supernatant. **B** The expression of PD-L1 in 4T1 cells. We prepared single-cell suspensions of mouse spleen cells and cocultured them with supernatant for 3 days. Then, one fraction of the cells was labelled with panel 1, PE-CD3, FITC-CD4 and APC-CD8, and panel 2, PerCP Cy5.5-CD3 and PE-CD279 (PD-1). **C** The percentages of PD-1^+^ and CD8^+^ T lymphocytes were analysed by flow cytometry. Total RNA was isolated from the other fraction of cells. After cDNA was synthesized, the expression of **D** Th1 cytokines (IL-2 and INF-γ), **E** Th2 cytokine (IL-10), **F** chemokine (CCL5) and **G** cytotoxicity-related genes (granzyme B and perforin) was analysed by real-time RT-PCR and normalized to β-actin (*n* = 4/group). The data are presented as mean ± SEM. *p < 0.05, **p < 0.01, ***p < 0.001, ****p < 0.0001 versus control; ^#^*p* < 0.05, ^##^*p* < 0.01, ^###^*p* < 0.001, ^####^*p* < 0.0001 versus rAd.Null. One-way ANOVA followed by Bonferroni post hoc tests was used.

To evaluate the immune stimulation effect of rAd.GM and rAd.Null in vitro, we infected 4T1 cells with rAd.GM or rAd.Null. The results showed that 4T1 cells infected with rAd.GM produced high levels of GM-CSF protein and was secreted into the extracellular medium (Fig. 5A). Interestingly, oncolytic viruses, especially rAd.GM increased PD-L1 expression in 4T1 cells (Fig. 5B). To observe the immune activation effect of oncolytic adenovirus, we prepared lysis supernatant of oncolytic adenovirus-infected 4T1 cells and cocultured with mouse splenocytes for 3 days. We found that the lytic supernatant increased the percentage of PD-1^+^ and CD8^+^ T lymphocytes in splenocytes and that rAd.GM-transduced cells performed better than rAd.Null-transduced cells (Fig. 5C). Furthermore, oncolytic virus treatments, especially rAd.GM, significantly increased the expression of Th1 cytokines (Fig. 5D) and cytotoxicity-related genes (Fig. 5G) and decreased the expression of Th2 cytokines (Fig. 5E) in splenocytes compared with the control. Moreover, rAd.GM also upregulated the expression of chemokines (Fig. 5F) that can recruit more T cells.

The same experiments were performed using EMT-6 and MDA-MB-231 cells. We found that oncolytic viruses, especially rAd.GM, increased both PD-L1 expression in EMT-6 cells and the percentage of CD8^+^ T cells in lymphocytes (Supplementary Fig. 1A). A consistent trend was also observed in MDA-MB-231 cells (Supplementary Fig. 1B). Similarly, oncolytic virus treatments, especially rAd.GM, steeply increased the expression of interleukin (IL)-2 and granzyme B and decreased the expression of IL-10 in EMT-6 (Supplementary Fig. 1C) and MDA-MB-231 (Supplementary Fig. 1D) cells.

The abovementioned results indicated the immune activation and tumour lysis effects of oncolytic viruses and the imperative for combination with aPC.

## Discussion

ICIs are becoming increasingly interesting in the field of cancer treatment, especially for non-small cell lung cancer and melanoma [20, 21]. Tumour cells can upregulate the expression of immune checkpoint molecules and related ligands and inhibit the activation of T cells, causing immune escape. ICIs can prevent this immune escape phenomenon and achieve antitumour effects. However, there are always many patients who are resistant to single ICI therapy due to low levels of inflammatory cell infiltration in the TME, including TNBC patients [21–24].

In this study, aPC single therapy slightly inhibited tumour growth and reduced metastasis in the lung, but the overall survival rate was not significantly improved. Accumulating reports have confirmed that T cell infiltration and activation towards CD8^+^ T cells are important factors affecting the efficacy of immunotherapy [25, 26]. The efficacy of immunotherapy is also related to the recruitment of TAMs and Tregs. The same phenomenon occurred in our results: aPC therapy did not effectively activate T cells, increasing the proportion of CD8^+^ and memory T cells and allowing tumours to express higher levels of Th1 inflammatory factors and killer cytokines, which may be the reason for the poor treatment efficacy. Moreover, the low immunogenicity of TNBC itself is a limiting factor. Therefore, an effective combination therapeutic strategy is needed to evoke the host immune environment.

Oncolytic viruses are regarded as the most promising therapies in immunotherapy because of their ability to replicate in tumour cells, directly dissolve tumours and release antitumour antigens to activate the host’s immune response. GM-CSF is a widely used therapeutic gene in oncolytic viruses. The oncolytic virus drugs T-Vec and Pexa-Vec, which carry the GM-CSF gene, were authorized by the Food and Drug Administration in the United States and have a significantly higher overall response rate in treated patients than in the control groups in many clinical trials [27, 28]. Our experiments in vitro confirmed that rAd.GM can lyse tumour cells, secrete high levels of GM-CSF and increase the level of cytotoxic T lymphocytes in the coculture system. In the abovementioned results, the immune activation effect of rAd.GM is stronger than that of rAd.Null in vitro. In animal experiments, oncolytic viruses significantly inhibited tumour growth, prolonged survival time and promoted tumour cell apoptosis. The crucial takeaway is that oncolytic viruses can effectively activate T cells in situ, systemically change the ratio of CD8^+^ to CD4^+^ T cells, increase the recruitment of memory T cells and reduce the ratio of TAMs and Tregs, leading to a transition in the immunosuppressive TME of TNBC. The therapeutic effect of oncolytic viruses carrying the GM-CSF gene is significantly stronger than that of control oncolytic viruses because rAd.GM can upregulate the expression of chemokines and Th1 cytokines more impressively than rAd.Null in tumours. However, it has been shown that oncolytic virus therapy can upregulate the expression of immune checkpoints and affect antitumour efficacy [16]. Similar results were also found in our study. We found increased PD-L1 and CTLA-4 expression in local tumour tissues after oncolytic virus treatment. Therefore, the combination of oncolytic viruses and aPC is a reasonable strategy with dual beneficial effects in immunotherapy.

The curative effect of the combined treatment groups was undoubtedly the most significant in our study, and both the tumour inhibition and the improved survival rate were better than those in the other monotherapy groups. Moreover, the combination treatment groups prevented lung metastasis, and approximately 20% of animals showed zero metastasis, which was not found in other treatment groups. In addition, we found that the combined treatment groups showed higher T cell activation than the oncolytic virus monotherapy groups, which strongly changed the proportion of CD8^+^ to CD4^+^ T cells. It is clear that TAMs are pivotal factors in tumour progression and are tightly associated with predicting immunotherapy [29]. In our study, TAMs were highly enriched in untreated TNBC tumours and tended to express the M2 phenotype, which indicates immunosuppressive conditions. Combination therapy can effectively reduce the proportion of TAMs, and it can cause the polarization of macrophages from the M2 to the M1 phenotype, thereby regulating the TME. Furthermore, combination treatment can upregulate the expression of cytotoxicity-related genes and regulate the Th1/Th2 balance, increasing the expression of chemokines to augment the density of T cell infiltration in tumours. Combination therapy of oncolytic viruses and aPC, particularly with rAd.GM and aPC, can reverse the tumour immunosuppression microenvironment of TNBC in various ways and have a synergistic antitumour effect.

The multiplex immunofluorescence analysis technique was used in this study to evaluate the TME. The results showing the effects on CD8^+^ T cells, memory T cells, Tregs, PD-L1 and CTLA-4 are consistent with the immune cell subset analysis in peripheral blood and spleen performed with flow cytometry, which is a reverification of the mechanisms identified from the periphery to the local part of the tumour. Multiplex immunofluorescence makes up for the limitations of flow cytometry in the analysis of immune cells, such as the small proportion of TAMs and the difficult isolation of infiltrating lymphocytes in the tumour. Multiple targets in a cell or tissue sample can be detected simultaneously in situ, and high signal-to-noise ratio images and accurate batch quantitative analysis can be obtained. These analyses can comprehensively, systematically, intuitively and scientifically present the situation of the TME, which is of great significance to the study of the occurrence and development of diseases, mechanistic research and curative effect evaluation.

It has been reported that some combination therapy can abrogate tumour development, but our study did not show this effect, which may be due to the differences in tumour type, animal models, combination strategies and treatment schedules. Goff and colleagues [30] combined Newcastle disease virus, anti-PD-1 and anti-CTLA-4 with radiation to achieve significant tumour freedom, which was largely due to 20 Gy radiation exposure. Deng and colleagues [31] used the CSF-1R inhibitor PLX3397 as one of the elements in combined therapy. In addition, their study established an MC38 colon cancer model whose immunogenicity is relatively high. In contrast, we treated 4T1 breast cancer cells, which are refractory to immunotherapy and show low immunogenicity. Previous studies on combination therapy of 4T1 cells have not shown complete regression of the tumour [16, 32, 33]. Kim’s [18] team used Pexa-Vec combined with ICIs to achieve 3/8 tumour free on Renca subcutaneously implanted tumour mouse model when the tumours reached 50 mm^3^, in contrast to our orthotopic transplanted tumour mouse model with treatment initiated when the tumours grew to 100 mm^3^. In addition, another reason our combination therapy did not completely regress the tumours may be related to the antibody dose and treatment schedule. Severe toxicity and side effects of ICIs in clinical trials have been reported [34]; therefore, we chose a lower dose (anti-PD-L1, 10 mg/kg; anti-CTLA-4, 4 mg/kg), not 20 mg/kg or 40–275 mg/kg in the mice [31, 35, 36], as in other experiments. The treatment schedule is closely related to the immune response [37]; therefore, the optimal time interval and frequency of drug injection may need further study. Although our study did not abrogate 4T1 tumour development, combined therapy significantly prevented lung metastasis and achieved remarkable therapeutic effects with fewer side effects.

Moreover, the current study included only a single mouse model (4T1), although the tumour growth and metastatic spread of 4T1 cells in BALB/c mice very closely mimicked human breast cancer, limiting generalizations. Diverse TNBC or other tumour implant models should be established to verify the therapeutic effect of rAd.GM + aPC.

In short, in this study, we provide a mutually reinforcing strategy for clinical immunotherapy to treat TNBC by combining oncolytic viruses with ICIs. Combination therapy can significantly inhibit tumour growth, prolong survival and prevent lung metastasis. Oncolytic adenoviruses can reprogramme the immunosuppressive TME, while ICIs can prevent immune escape after oncolytic adenovirus therapy by reducing the expression of immune checkpoint molecules. This is the first report showing that oncolytic adenoviruses, especially rAd.GM, combined with aPC may be a novel strategy for treating TNBC patients.

Although the complementary combination therapy of oncolytic viruses with ICIs offers a ray of hope for TNBC patients, most studies are still in its infant stage. The route of drug injection, the sequence and dose of the combination therapy and the selection of the most suitable volunteers need further rational design. In addition, the selection and preparation process of oncolytic viruses, the expression of immune checkpoints and biomarkers for predicting efficacy need to be further investigated. We believe that, with the progress of tumour immunology, oncolytic viruses combined with ICIs will have broader prospects in the treatment of TNBC.

## Supplementary information

Supplementary Figure 1 legend

Supplementary Figure 1

## Data Availability

All data generated and/or analysed during this study are included in this published article. Data sharing is not applicable to this article, as no data sets were generated or analysed during the current study. However, the data that support the findings of this study are available from the corresponding author upon reasonable request.
