# Supplementary material for: Oncolytic adenoviruses synergistically enhance anti-PD-L1 and anti-CTLA-4 immunotherapy by modulating the tumour microenvironment in a 4T1 orthotopic mouse model
Source: Cancer Gene Ther. 2021 Sep 24;29(5):456–65. doi: 10.1038/s41417-021-00389-3 (PMC9113929; doi:10.1038/s41417-021-00389-3)
Supplement: Supplementary file 1 — Supplementary Figure 1 legend [file 41417_2021_389_MOESM1_ESM.docx]

Supplementary Figure 1. Immune activation effects of oncolytic virus therapies in EMT-6 and MDA-MB-231 cells.

EMT-6 and MDA-MB-231 cells were infected with oncolytic viruses for 48h. We collected the cells and supernatant separately, and then add the mouse spleen cells or human lymphocytes to the supernatant for co-culture. (A) The expression of PD-L1 in EMT-6 cells and the percentages of CD8+ T lymphocytes in mouse splenocyte. (B) The expression of PD-L1 in MDA-MB-231 cells and the percentages of CD8+ T cells in human lymphocytes. Total RNA was isolated from the mouse splenocytes and human lymphocytes separately. After cDNA was synthesized, the expression of granzyme B, IL-2 and IL-10 in EMT-6 cells (C) and MDA-MB-231 cells (D) co-culture was analyzed by real-time RT-PCR and normalized to β-actin. The data are presented as the means±SEM. * p<0.05, ** p<0.01, *** p<0.001, **** p<0.0001 versus control; # p<0.05, ## p<0.01, ### p<0.001, #### p<0.0001 versus rAd.Null. One-way ANOVA followed by Bonferroni post hoc tests was used.
