# Supplementary figures and images for: Oncolytic adenoviruses synergistically enhance anti-PD-L1 and anti-CTLA-4 immunotherapy by modulating the tumour microenvironment in a 4T1 orthotopic mouse model
Source: Cancer Gene Ther. 2021 Sep 24;29(5):456–65. doi: 10.1038/s41417-021-00389-3 (PMC9113929; doi:10.1038/s41417-021-00389-3)

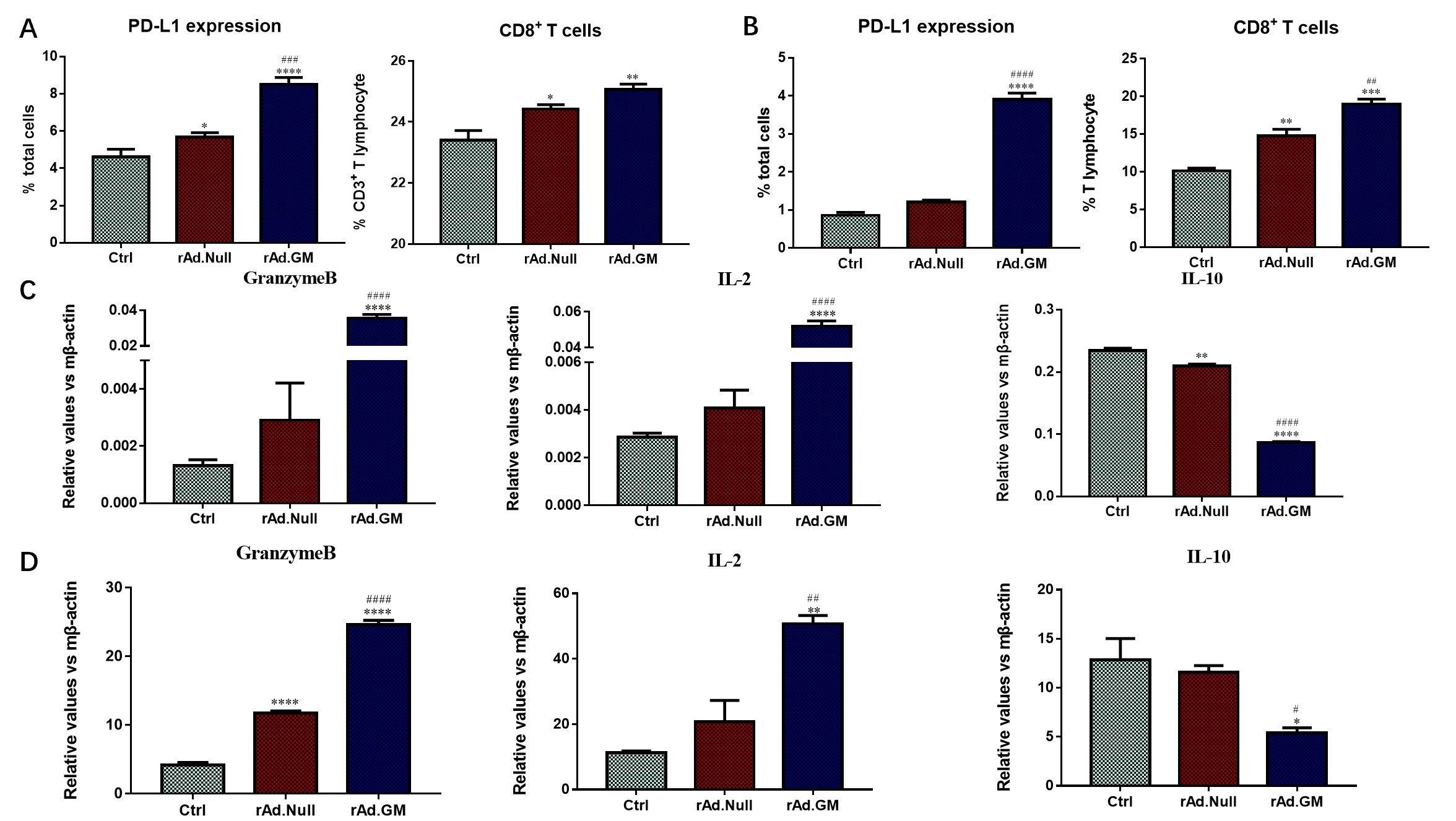

Supplement: Supplementary file 2 — Supplementary Figure 1 [file 41417_2021_389_MOESM2_ESM.jpg]
